# Potential survivable niches for microbial life on the lunar south pole

**DOI:** 10.1126/sciadv.aec0811

**Published:** 2026-08-19

**Authors:** Prabal Saxena, Stefano Bertone, Heather V. Graham, Natalie M. Curran, Aaron B. Regberg, Andrew Needham, D. E. (Betsy) Pugel, Noah E. Petro

**Affiliations:** ^1^ NASA Goddard Space Flight Center, Greenbelt, MD 20771, USA.; ^2^ University of Maryland, CRESST II, College Park, MD 20742, USA.; ^3^ National Institute for Astrophysics (INAF), Astrophysical Observatory of Turin, 10025 Pino Torinese, Italy.; ^4^ Catholic University of America, CRESST II, Washington, DC 20064, USA.; ^5^ NASA Johnson Space Center, Houston, TX 77058, USA.

## Abstract

Most lunar surface conditions are incredibly harsh for microbial survival. High ultraviolet radiation, temperatures, and energetic particle radiation limit survival over most unprotected lunar surfaces, particularly in equatorial regions where all previous crewed exploration occurred. However, whether these harsh conditions are widespread at lunar poles has not been examined considering topographical effects. Here, we show that recent microorganism survivability data and lunar surface remote sensing reveal likely survivable niches in lunar polar regions. Analysis of topography and latitude-driven surface conditions using remote sensing data and high-resolution illumination models indicates the lunar south pole has substantial regions with persistent low temperatures and ultraviolet flux. Comparing these conditions to survivability data of specific microorganisms, we find that significant lunar polar areas likely have surface conditions amenable to microbial survival. Our findings suggest that lunar polar regions may be less hostile to microbial survival than previously assumed. This does not encompass growth likelihood, but survival in a cryptobiotic state where growth would be possible if habitable conditions were present. Potential microbial survivability at lunar poles is particularly significant given many examined microbes will likely be transported to the Moon during crewed lunar south pole exploration planned in numerous near-term missions. Thoughtfully planning exploration and tracking its impact is key to limiting and understanding potential unintended life transfer to the Moon.

## INTRODUCTION

The near-term plans for a sustained surface presence at the lunar south pole represent a significant shift in human space exploration. This unprecedented plan for habitation at a location outside low earth orbit offers unique opportunities for science, exploration, resource identification and extraction, and engagement of the general public with space. However, such plans would also inevitably lead to an anthropogenic influence on the environment surrounding the habitat and exploration zones, thus affecting scientific questions including those related to prebiotic molecules and other volatile organic compounds (*1*, *2*) that can be posed at these locations.

The lunar south pole has unique properties relative to the rest of the Moon, some of which enable the presence of surface/near-surface water ice, which is part of the motivation for the heightened interest in the region. Low Sun elevation angles at the lunar south pole due to the Moon’s orbit and obliquity and to regional topography result in portions of the surface that receive significantly attenuated amounts of solar radiation. These factors are what enables the presence and stability of water ice (*3*–*5*) and generally lead to low amounts of ultraviolet (UV) light, low temperatures, and low energetic particle flux in regions at the south pole relative to the rest of the Moon.

These unique properties present an interesting test case with respect to the survival of life—the present-day Moon and exposed space have generally been regarded as extremely harsh environments for microbial life (*6*–*8*). A previous study (*8*) found that UV light exposure and high temperatures are the key factors in making space environments the most hostile for microbial life and that the likelihood of survival of microbial life left on the lunar surface by previous exploration in equatorial regions of the Moon was deemed to be low. However, the model did not include the effects of topography; although these effects may be muted at regions with potentially high Sun elevation angles (such as equatorial regions), in polar regions, topography plays a significant role in local conditions relevant to microbial survival.

This is particularly important given the context of the evolving understanding of the survival of microbes exposed to space environments. Microbial survivability in space environments is not only merely conditional on the external stresses it is exposed to but also to the microorganism’s response mechanisms including structural, metabolic, genetic, and even ecological features (*9*–*11*). Intentional and serendipitous space exposure (*12*–*16*) and laboratory experiments (*17*–*19*) suggest an evolving understanding of microbial survivability in space. The potential that certain microbes may be capable of surviving space conditions that may be relevant to the lunar south pole motivates a joint analysis by our team of observed/inferred surface conditions at the lunar south pole versus survival bounds of relevant microbes.

We focus on three bacterial genera and two fungal genera; four that are representative of the ubiquitous of opportunistic microbes likely to be transported to the lunar surface as part of human exploration, as well as an extremophilic bacteria that has been shown to be particularly well adapted to the harsh conditions of space environments and has been cultured in NASA clean rooms. We consider survival bounds of species within these genera to determine a plausible phase space for the two most critical biocidal factors on the lunar surface—UV radiation and temperature. These genera are resistant to vacuum conditions (*8*–*10*, *12*, *20*–*22*), temperature extremes, and energetic particle radiation, with specific discussion of the examined microbes’ survivability under these conditions given in the Supplementary Materials. These are all microbes that are commonly found in crewed space habitats (*20*, *23*–*31*), which makes them more likely to be transferred to the lunar surface given that such a habitat would accompany human exploration of the surface. Because there is a well-established correlation between solar-sourced particle radiation and UV radiation (*8*), we use UV exposure and temperature as proxies to map local conditions at the lunar poles and identify specific locations where microorganisms could survive for at least 1 Earth day. This timescale is chosen because 1 Earth day is greater than the longest time between consecutive extravehicular activities (EVAs) previously conducted on the moon. Microbial survivability between consecutive EVAs has the potential to affect scientific operations by increasing the organic contamination baseline in permanently shadowed regions (PSRs) (*32*) and confounding our search for prebiotic molecules and other volatile organic compounds that may be concentrated in these locations (*33*, *34*).

This survival estimate leverages recent remote sensing products, which allowed us to characterize surface conditions at highly resolved spatial scales, allowing us to consider the shadowing effects of local topography. Temperature data are based on measurements by Lunar Reconnaissance Orbiter’s (LRO) Diviner (*35*) that yields seasonal temperatures at the scale of 240 m, whereas regional UV (≤320 nm) fluxes can be estimated on the basis of average illumination maps with a pixel scale of 60 m based on topography derived from LRO Lunar Orbiter Laser Altimeter (LOLA) measurements (*36*). We then analyzed UV fluxes at finer spatial scales for several Artemis III candidate landing sites (*37*) by ray-tracing solar illumination to updated LOLA-based topography maps (*38*) with a pixel scale of 5 m/pix. Artemis III is planned to be the first time humans will explore the lunar south pole, as part of NASA’s Artemis campaign to reestablish a human presence on the Moon in preparation for future human exploration of Mars. Last, we briefly discuss the impact of submeter-scale topographic features due to both anthropogenic and nonanthropogenic sources at relevant polar exploration sites. We find that both lunar poles likely have significant regions of potential microbial survivability—microbes examined may be able to survive in these regions (including in their PSRs) in a cryptobiotic state, although we do not believe that growth is likely to occur unless habitable conditions are met (see Discussion later in study). This finding has significant implications for both science and exploration requirements and updates prevailing views that have been influential on policy (*39*).

## RESULTS

### Survivability bounds of key relevant microbes

Selection of specific microorganisms for our analysis is based on a number of different factors that reflect important considerations regarding both their ability to survive on the surface at the lunar south pole and likelihood of their delivery to the surface. Some of these particular microorganisms have demonstrated survival in space (*20*, *24*, *25*, *40*, *41*) or have shown they may proliferate in a potential surface payload cabin. Another key factor is how cosmopolitan (common and widespread) a particular microorganism may be. We note that our choices are a small subsample of the diverse range of microbes that may be transferred to regions of exploration and that they may not include relatively common microbes that are also highly adapted to survival on the lunar poles.

Another key distinction in our analysis is our focus on how survivable a particular niche may be versus how habitable it may be—the threshold for growth is higher than that of survival (*42*). Survival implies that the cell is at least in a dormant state, behaving as a persister cell, is viable but not may not be culturable or has formed a spore (*43*). Cells in any of these survival states can return to more active metabolism when conditions change. We also note that cells may be dead but still persist in the environment because they persist in the environment as potential sources of contamination (*44*). Growth requires active metabolism and potential reproduction. Reproduction can be observed by cultivation, whereas active metabolism can be observed as respiration, enzymatic activity, translation, transcription or changes in membrane potential. Moribund or dead cells will not perform any of these functions and likely will have damaged intracellular biomolecules and phosphorylated membrane lipids. Stains can be used to differentiate cells in this variety of metabolic states (*45*, *46*).

However, even survival is highly salient to exploration and science—such survival would offer a previously unidentified example of the existence of life on another planetary body in the corresponding local environment, is a precondition for potential growth, and would also be critical to take into account when conducting astrobiological studies or assessing the local human health environment. The microbes [*Bacillus* (*47*), *Deinococcus* (*48*), *Staphylococcus* (*49*), *Aspergillus* (*50*), and *Fusarium* (*51*)] selected for this study are extremophiles capable of surviving the conditions expected at some lunar locations and/or are cosmopolitan organisms commonly detected in crewed spacecraft.

The microbes are given in Table 1 and are representatives of the microbiome that will likely accompany human space exploration. Common occurrences and motivation for inclusion are described in the second column. We list maximum growth temperatures for each microbe as opposed to survival temperatures to use conservative high-temperature bounds for survival for a particular microbe and given that relevant studies more commonly measure temperature limits of growth versus survivability. These microbes may survive at higher temperatures, although temperatures in the lunar poles are generally lower than these values. The final column lists empirically derived UV lethal dose values (for each corresponding life cycle stage of a microbe) that we use as a maximum fluence (time integrated flux) above which there is no survival assumed for the particular microbe. Details and assumptions related to how we obtained or estimated these values, as well as related background, are given in Materials and Methods. We do not include values related to vacuum conditions, energetic particle flux, additional radiation sources such as galactic cosmic rays (GCRs) and Earthshine or low temperatures in the table given that they are likely to have negligible relative impact on survival of the chosen microbes over our timescales of interest (see Materials and Methods for analysis of each of these processes).

**Table 1.** Survivability assessments for microbial candidates. Lethal UV doses are given on the basis of the environment of the experiment [irradiation conducted in air or on a surface; relative humidity (RH) amount if in air] and the status of the organism with respect to whether cell in the experiment is in a vegetative (veg) or spore state. All growth values are for the vegetative state. ISS, International Space Station. Bold signifies which values for each category were used in the analysis.

Microbe	Places commonly found/motivation for inclusion	Max growth temperature dry (K)	UV lethal dose (6 log reduction) in J/m^2^
*Bacillus* (*subtilis*)	Very common human-associated heat-resistant mesophile sporeformer	**328** (*120*)	84 (veg, air/low RH) (*121*), **410 (endospore, surface)** (*122*)
*Deinococcus* (*radiodurans*)	Radiation resistant, demonstrated survival in exposure to space	**331** (*20*)	**2280 (veg, surface)** (*123*)
*Staphylococcus* (*aureus*)	Very common heat-resistant human-associated mesophile	**324** (*124*)	**300 (veg, surface)** (*125*)
*Aspergillus* (*niger*)	Radiation resistant, common ISS contaminant	**315** (*126*)	27,000 (veg, surface) (*127*), **30,560 (spore, surface)** (*18*)
*Fusarium* (spp.)	Common heat-resistant soil fungus and ISS contaminant	**314** (*128*)	6720 (veg, surface) (*127*), **3360 (spore, surface)** (*127*)

Maximum growth temperatures for the five microbes span a range of less than 20 K (the selected fungi are less tolerant to high temperatures than the bacteria) and, as detailed in the next section, are all above modeled maximum summer temperatures in the lunar south pole for nearly the entire region. The estimated UV lethal dose fluence, however, varies to a much greater extent. Although bacteria such as *Bacillus* and *Staphylococcus* are inactivated at relatively low values, the selected fungi (*Aspergillus* and *Fusarium*) require fluences for inactivation that are orders of magnitude greater than the bacteria. In microbiology, inactivation refers to a set of environmental factors that permanently render an organism incapable of reproducing (*52*, *53*). Given that temperatures at the lunar poles are rarely high enough to be biocidal and that low temperatures are used to preserve microbes on Earth, UV radiation appears to be the most effective biocidal factor in those regions. Therefore, we focus on mapping UV fluences in the lunar polar regions to identify regions where microbes may survive. We also exclude the few locations where temperatures exceed thresholds needed to inactivate our target organisms.

### UV and temperature environments at the lunar south pole

**Fig. 1.**
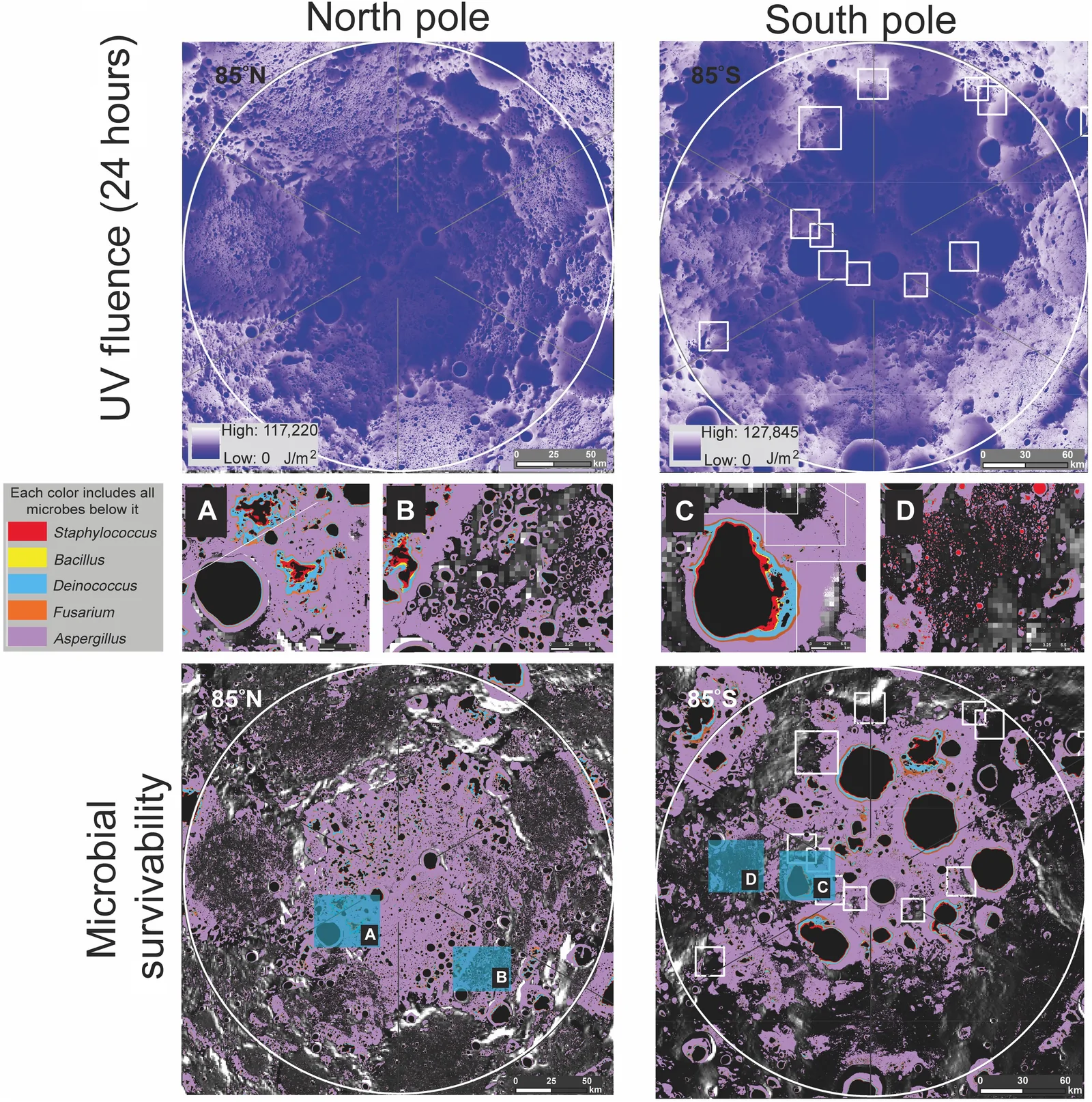
Microbial survivability in the polar regions of the Moon. Top panels show the UV fluence over a 24-hour period in the polar regions of the Moon. Bottom panels show areas where each microbe may be able to survive based on corresponding limits related to the direct integrated UV fluence and maximum summer temperature (with PSRs, given in black, as survivable for all microbes under this scenario). (**A**) North pole enlarged region showing survivability for multiple microbes in the vicinity of larger PSRs. (**B**) North pole enlarged region displaying survivability patterns for multiple microbe species interspersed with areas of lower survivability. (**C**) South pole enlarged region near the De Gerlache region showing the survivability of multiple microbes surrounding the PSR. (**D**) South pole enlarged region showing survivability patterns for either multiple microbe species or only *Aspergillus* interspersed in large area of lower survivability. White squares on the bottom panels show the Artemis III candidate regions. Round circles on each of the large panels show the 85°N or 85°S.

**Fig. 2.**
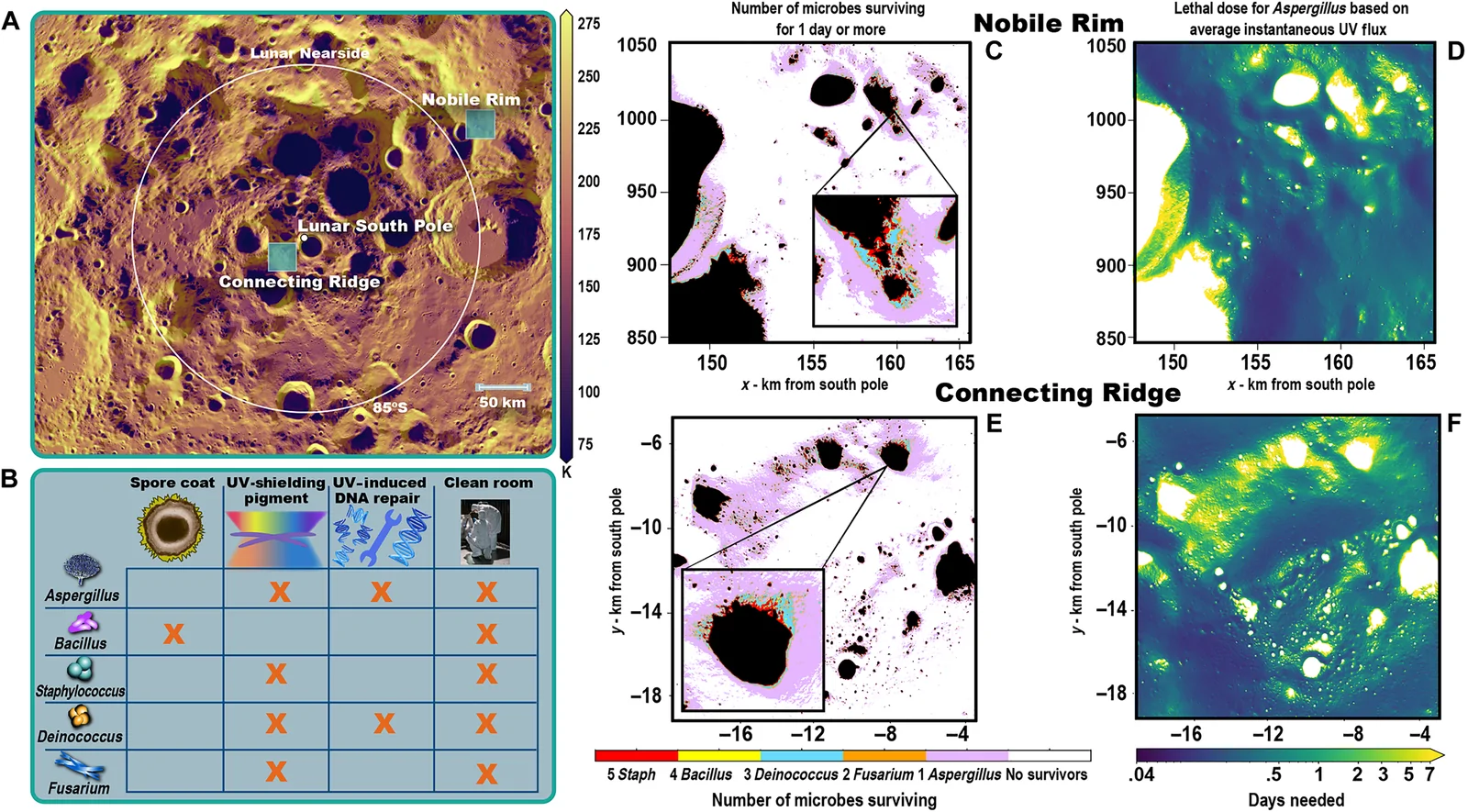
Microbial survival mechanisms and lunar surface conditions at Artemis III sites. (**A**) Maximum summer temperature map showing the thermal environment at the lunar south pole, with cooler temperatures (darker colors) providing more favorable conditions for microbial survival. (**B**) Survival mechanisms table showing key adaptations of the five examined microbes, including spore formation, UV-protective pigments, and DNA repair capabilities, that enable survival under harsh lunar conditions, as well as clean room prevalence, which makes transfer more plausible. (**C**) Number of microbial species surviving after 1 Earth day at the Nobile Rim region, showing the spatial distribution of survivability with up to five species surviving in the most favorable locations. Similar to Fig. 1, the larger the number includes all the species listed below the color. (**D**) Similar to (C), the number of microbial species surviving after 1 Earth day at the Connecting Ridge region, demonstrating areas where multiple species can survive simultaneously. (**E**) *Aspergillus* survivability duration map for Nobile Rim showing time periods (in days) that this most resilient species can survive, with maximum survival times of at least 7 days in optimal locations. (**F**) Similar to (E), *Aspergillus* survivability duration map for the Connecting Ridge region, indicating extended survival periods with some areas supporting survival for 7+ days.

With the survivability limits for selected microbes from Table 1, based on temperature and UV dose, we can filter for potentially survivable regions of the lunar poles at different spatial scales. A number of datasets were combined to produce regional survivability maps for the north pole and south pole of the Moon. The regional polar maps (*54*) that are used to approximate UV fluence calculate averaged instantaneous UV flux over a lunar day using fractional solar visibility based on the LOLA topography (*36*) and an analytical formulation (*55*) of illumination for flat surfaces based on orbital and latitudinal properties. Temperature data for our regional maps are based on the maximum summer temperature from LRO Diviner data (*35*). The top two maps in Fig. 1 show regional UV fluence polewards of 85° for the north pole and south pole of the Moon. These maps provide the integrated UV light fluence over 1 Earth day (24 hours) considering direct illumination, based on the average instantaneous flux at the surface. They are meant as a qualitative overview of potential survivability because their incorporation of topography is at coarser spatial resolution (see Materials and Methods for details). Although fluences are similar within an order of magnitude to more accurate high-resolution maps (Fig. 2, C to H), the implicit assumption of flat terrain with regard to local topography in the fractional illumination calculations results in underestimates in more poorly illuminated regions and overestimates in highly illuminated regions. Nevertheless, these maps provide important guidance for potential survivability of different microbes over relevant timelines and can inform selection of smaller areas for more accurate modeling.

Using threshold criteria of a maximum summer temperature under the maximum growth temperature and an integrated average instantaneous UV fluence that does not exceed lethal dose values based on Table 1, the bottom two maps in Fig. 1 display an estimate of regions where the microbes of interest likely could survive for at minimum 1 day (24 hours). The top panels of Fig. 1 show the significant variability in integrated UV fluence over 1 day near the poles, which spans from roughly 1 MJ/m^2^ in the most highly illuminated regions to no direct UV illumination within PSRs. Fluence also generally decreases towards the pole due to lower Sun elevation angles. Because temperature is highly correlated with UV flux, survivability to temperature broadly follows the trend related to the lower fluence. Modeled conditions in Fig. 1 show that *Aspergillus* exhibits the greatest potential for survivability in polar regions with respect to the criteria we identify to be most important, likely due to repair mechanisms that lend exceptional resilience to UV fluence. There are additional regions of survivability for other combinations of the microbes, but they are significantly smaller and generally proximate to PSRs—some of these regions are shown in the magnified maps in the middle of the image, with illustrative subregions chosen from the respective pole. These maps are simplified but show general trends of survivability that provide guidance for selecting specific regions where survivability is modeled more accurately by considering higher-spatial-resolution properties—details behind both sets of maps are given in Materials and Methods. Each of the regional maps indicates there are potential regions of survivability in the context of joint UV flux and high-temperature limits for all the microbial species in this study at the north pole and south pole of the Moon. As we note in Discussion, a more thorough survivability analysis would incorporate higher-spatial-resolution data and would require additional study of multiple additional and joint factors that may be relevant to survivability through careful experiments and modeling—this study examines survivability in the context of currently understood key factors for survival on the lunar surface exposed to open space.

Although regional maps provide a large-scale overview of microbial survivability, the coarser resolution at which they model topography driven shadowing may miss out on potential survivability at finer spatial scales. We thus model varying illumination conditions at sites relevant to Artemis exploration by ray tracing on recent high-resolution LOLA-based topography (*38*) (details given in Materials and Methods). We compare seasonal UV fluences over timescales of interest masked with maximum surface temperature values (derived from lower-resolution Diviner maps at 240 m/pix) to microbial thresholds from Table 1 to produce survivability maps similar to but more detailed than the regional polar maps shown in Fig. 1. These maps are shown in Fig. 2 and display the number of surviving microbes at selected Artemis III Candidate Landing Regions after 1 Earth day in August 2026 (i.e., close to the Sun equinox at the Lunar south pole) and the survivability of *Aspergillus* (the most resilient of the reference microbes from Table 1) over timescales ranging from a few hours to at least 7 Earth days, for reference. Critically, for these maps, we only model direct illumination because scattered light (particularly in non-PSR regions) has a negligible effect on total survivability timescales and extents (see Materials and Methods for more details). We do, however, include modeling of scattered light for the case study of a PSR in the De Gerlache region as it is likely to control survivability timescales. In the Materials and Methods section, we also analyze additional potential sources of UV flux, such as all-sky Lyman Alpha flux and Earthshine, and find that they are likely to have a minimal contribution to survivability limits given the timescales we are examining.

Figure 2A shows the maximum summer temperature close to the Lunar south pole, which is used in all maps as a limit on survivability. Below this map is a table that shows key survival adaptations or factors related to the five microbes of interest. To the right of these images are first the maps depicting the survivability of the different microbes for 1 day and then maps showing the average time needed to accumulate a lethal dose of UV flux for *Aspergillus*. These maps differ from Fig. 1 in that we model illumination by explicitly using ray tracing on recent high-resolution LOLA-based topography. Both selected areas—those in the Nobile Rim and Connecting Ridge regions (with De Gerlache listed in Table 1)—contain significant places where microbes may be able to survive. Of note is that these potentially survivable areas are not just limited to those adjacent to larger PSRs and, in many cases, are nearby areas that receive high levels of illumination. *Aspergillus* is the organism that may be able to survive in the largest number of places given our analysis. It may be able to survive in 2 to 9% of the mapped (non-PSR) surface area in the summer and 15 to 30% of the mapped area in the winter. PSRs are not probed by direct illumination models and thus are not included in these calculations, although they receive less radiation and experience lower maximum temperatures than other regions. Fractional areas of survival are roughly 4 to 7 times lower for *Fusarium*, 5 to 9 times lower for *Deinococcus*, and 11 to 30 times lower for *Staphylococcus* and *Bacillus*. The Nobile Rim 2 region contains the highest fraction of potentially survivable areas for the three hardiest microbes during summer and the Connecting Ridge region contains the highest area during winter for all of the microbes. Maximum survivable area and order of total area of survivability vary between all three regions depending on the microbe, for autumn and spring, although Connecting Ridge again contains the largest survivable area for the three hardiest microbes. The De Gerlache Rim region shows the lowest survivable area during summer, whereas Nobile Rim 2 has the lowest survivable area during winter—expectedly, survival rates peak in winter and are at a minimum in summer. Critically, all three regions have roughly 3% survivable area for *Aspergillus* for timescales of at least 7 days, indicating longer-term potential survivability (how long merits additional study) of some microbes across these regions of the south pole.

**Fig. 3.**
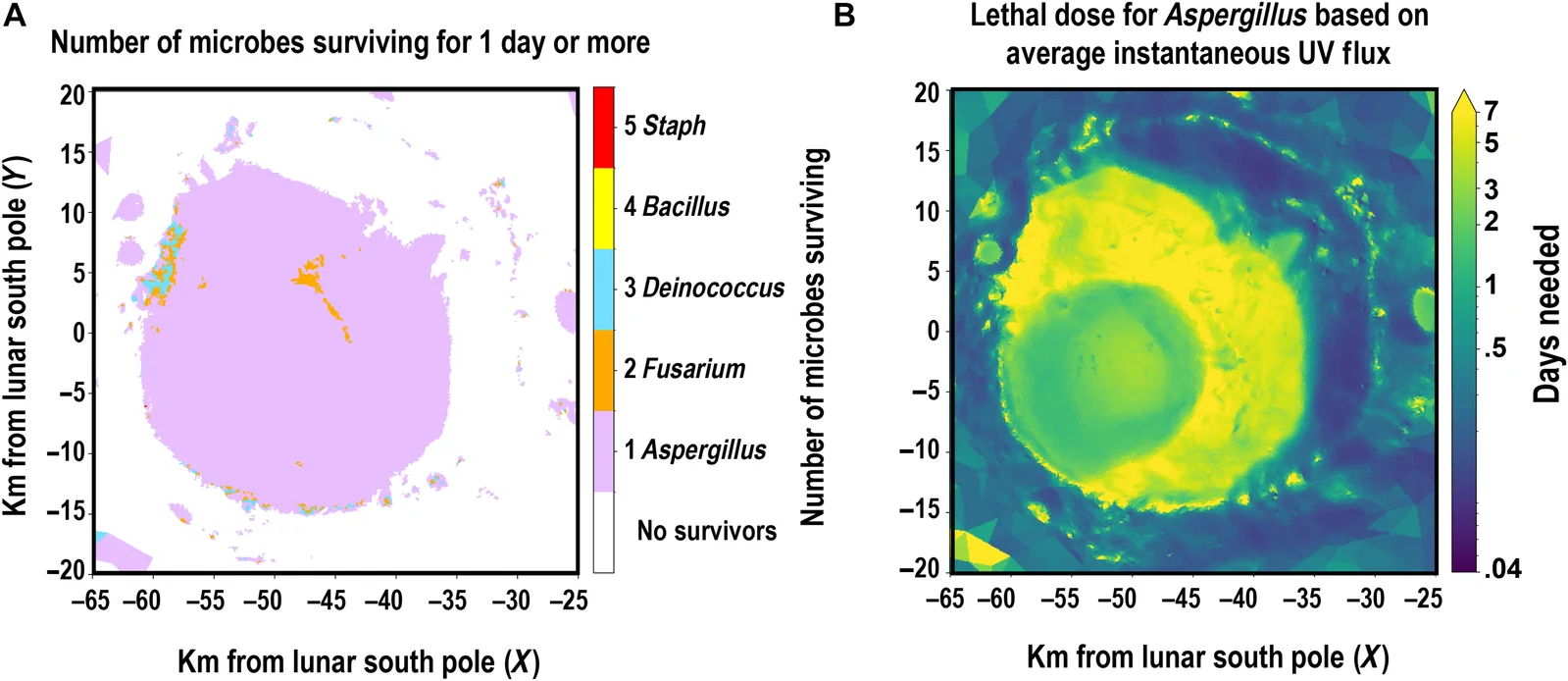
Impact of scattered UV light on microbial survivability within PSRs. Lunar PSRs never received direct sunlight because Sun’s elevation never exceeds their local horizon. However, flux scattered by nearby topography still reaches them. We model scattered UV flux within De Gerlache’s PSR to highlight its impact on survivability. (**A**) Microbial survivability map showing which species can survive at least 1 day within the PSR when accounting for scattered UV light, with the same color scheme as in Fig. 1 (*Aspergillus* in cyan showing the most extensive survival areas, followed by other species in decreasing order of survivability). (**B**) *Aspergillus* survival duration map showing survival time in days within the PSR, with the most favorable areas (yellow) supporting survival for the maximum modeled period of at least 7 days, whereas other areas show progressively shorter survival times (darker colors).

Fluxes within PSRs are set to 0 and masked out (i.e., black) in regional and high-resolution maps presented in Figs. 1 and 2 because we only model direct sunlight for those maps. These regions are the most favorable to survival due to lack of direct illumination and are likely to have their limits set by scattered light, which is modeled in Fig. 3. Total fractional areas of potential survival using our high-resolution maps, including separate estimates of PSR areas, are given in table S1. Because PSRs will be more heavily affected by scattered sunlight from neighboring illuminated surfaces—as an example, Fig. 3 shows survivability within De Gerlache’s PSR (88.5°S, 87.1°W) when modeling scattered fluxes using established methods (*56*, *57*). We assume surface albedo at UV wavelengths to be the same as for visible light. We show the impact of scattered UV light in limiting survivability areas for multiple microbes and the estimated lifetime for the most resilient species in our set, *Aspergillus*. The impact is mostly relevant within PSRs as scattered flux represents ∼1% of the direct solar flux in illuminated areas. Fractional values of survival in the region using this methodology are also given in table S1. Including scattered light does reduce the total fractional area of potential survival for microbes and the total time of potential survival within the PSR, but the fractional area of potential survivability is still much larger than in illuminated regions, as expected. All five microbes examined can survive in some regions of the PSR for periods longer than a week, which is broadly similar to the findings of other work (*58*) that examined survival of *Bacillus* in two PSRs for a specific season and comports with earlier works (*59*, *60*) that noted potential survival of microbes in PSRs. Our results are consistent with the assertion that lunar PSRs are, “one of the least biocidal environments in the solar system” (*58*). However, differences between these survival times, variations across different PSRs, and consideration of other processes that may operate on relevant timescales all motivate the need for higher-resolution spatial and temporal studies of these PSRs. Despite that, although our focus is on the general question of potential survivability, studies looking to analyze a more localized region in higher detail should incorporate scattered light, despite the significant additional computational expense.

Analysis of potential survivable niches in the context of UV fluence is relevant at even higher-spatial-resolution scales—recent Chang’e 3/4/5 mission results show the average depth-to-diameter ratios of smaller craters [*d*/*D* of ∼0.07 for craters of 1 to 10+ m (*61*) and *d*/*D* of ∼0.055 for craters of ⪅1 m (*62*)] in relatively high-latitude regions that suggest additional small-scale survivable niches are likely to exist in the polar regions. These two values of *d*/*D* would result in permanently shadowed craters on flat terrain polewards of 87°. Planned human exploration activity is likely to also create similar small-scale survivable features—although drill cores and large scoops can create shadowed regions at more equatorial latitudes, even boot prints, rover wheels, and small scoops are likely to leave shadowed depressions on flat surfaces at polar latitudes relevant to exploration (*63*).

## DISCUSSION

### Implications for lunar exploration

The unique properties of the lunar polar regions (both the south pole and north pole) with respect to exposure to sunlight suggest that there are likely widespread survivable niches for different microbial life in those regions. Vectors for delivery of microbes relevant to those examined in this study are likely to be persistent and frequent due to upcoming exploration (especially because Artemis III candidate landing sites are also candidates for other missions and a base camp)—venting from airlocks (*64*–*67*) and spacesuits that may release organics are also likely to release life, although filtration techniques may mitigate this somewhat. Although some of the examined microbes do exhibit survival in spite of clean room contamination control strategies, the presence of humans during exploration inherently increases the number and diversity of microbes that may be transferred because humans themselves are vectors for delivery, given that they carry millions of microbes (*68*, *69*) on their skin and even more in their bodies. We note that, now, there are no bioburden requirements for spacecraft bound for the lunar surface [see (*70*)]. Best practices to support science efforts motivate reevaluation of current contamination knowledge-driven planetary protection requirements for Category II PSRs (*71*). Our findings show that, for many investigations, it would be prudent to use more stringent contamination mitigation procedures (*1*). Although we view transfer during exploration as the most likely delivery mechanism of life to the polar regions, it is important to note other means of transfer of material to the lunar poles. For example, meteoritic transfer from outside the Earth-Moon system (*72*, *73*) may be able to deliver organics to the lunar surface whereas transfer of both meteorites (*74*, *75*) and molecules (*76*–*78*) from the Earth may be able to deliver organics and/or life, although, in all cases, survivability will be controlled by the dynamics of the delivery.

Because survival of microbial life may be plausible given our analysis related to the currently understood key factors, a natural question is whether there may exist habitable conditions that enable growth for survival candidates. We focused on survival as a less stringent precursor to growth partially due to our belief that the lack of a stable, dense atmosphere is likely to preclude a key growth factor, i.e., the existence of liquid bioavailable water at the surface. Although periods in the geological past (*79*) may have been habitable windows due to the brief existence of collisional atmospheres (*80*, *81*), the only potential present-day atmospheres (*82*, *83*) are likely to be highly transient and localized, although we note that anthropogenically generated atmospheres may be concurrent with delivery of microbial life. Concurrent potential delivery of organics that can serve as nutrients at the same time as delivery of microbial life (*67*) may be a growth enhancing factor, as may be modification of the surface to create trapped water that may episodically melt [see a previous work on potential short-lived subsurface lunar water (*84*) and fluidization studies on comets (*85*, *86*)], but both those mechanisms require additional study. Modification of the lunar environment due to human exploration that will inherently occur episodically but concurrently with potential transfer of life may result in novel processes relevant to these questions.

The plausibility of survival of microbial life in these regions is predicated on existing experimental work and modeling derived from existing remote sensing data. Additional study is needed in numerous areas for more refined analysis of potential survivable niches for microbial life—these additional studies should include experimental work that examines key additional properties of the lunar surface that may inhibit survival, the interactive effects of such properties, broader and higher spatial and temporal resolution studies of survival, and a more thorough and focused examination of work related to microbial survivability that has previously been carried out in a more general way. For example, although most regions do not likely have temperatures high enough for the biocidal interactive effects of the combination of simultaneous high temperatures (near or above growth limiting temperatures) and UV radiation (*8*) to become critical, this interaction may be true in some small pockets and additional experimental work for lunar analog environments would be illuminating. Understanding the number, diversity, and communities of microbes that may be released is another key area of future work—even dead microbes on the lunar surface may complicate astrobiological and other studies. Experimental UV light lethal dose studies that extend to lower survival fractions in lunar analog environments would also provide more accurate estimates. From the remote sensing perspective, higher-resolution digital elevation models (DEMs) of the lunar surface down to subcentimeter scales would enable more accurate modeling of key factors relevant to potential survivability—this is not limited to the poles as other natural features (e.g., pits and dikes) can generate niches at lower latitudes.

Understanding complicating effects of geology and other geophysical processes, surface modification due to human exploration and potential effectiveness of different mitigation techniques (*87*) are all also areas of potential future work. For example, models of impact gardening predict an average of equilibrium excavation and burial that would affect the top micrometer of the regolith on a timescale of roughly 10 years and the top centimeter on a timescale between 50,000 and 100,000 years (*88*, *89*)—although the time to affect microbes at these depths is longer than timescales of interest in this paper, such a process should be considered for longer-term survivability studies. Gardening of the regolith by human exploration is likely to impact a range of different depths depending on direct or indirect contact with the surface, and consequently emplacement of microbes may be at varying depths. Many of the processes that we have been able to ignore as negligible over this study’s timescales of interest may have nonnegligible impact over longer time periods and should be considered for relevant survivability studies (see Materials and Methods).

We note that studying such questions, and related ones such as those concerned with locations that may be survivable as well as limiting transfer, have applicability beyond the Moon and are an opportunity for science, not necessarily merely a hindrance. A survivable niche may not necessarily be a place to avoid during exploration but instead one to target for sample acquisition (potentially repeatedly) using a well-thought-out sampling strategy. A number of different airless bodies such as Mercury, Ceres, Asteroids, Comets, and Exoplanets that may have similar properties could also have potential survivable niches for microbial life. Last, understanding how these survivable niches may exist, how life may be transferred to them, and how human exploration may leave its mark all will serve as a key testbed for future potential human exploration of Mars, which likely has far more habitable environments.

## MATERIALS AND METHODS

The following sections contain information on the methodology and assumptions that contributed to the findings in the main body of the paper.

### Microbe properties: Assumptions and derivations

This section details assumptions and underlying derivations and data that were used to derive values in Table 1, which were then compared to lunar surface properties. As stated in the main text, the key factors examined in this study with respect to microbial survival were UV radiation and high temperatures. Vacuum conditions, energetic particle flux, and low temperatures were not included due to evidence from the literature they were not dominant or significant factors in inhibiting survival of the chosen microbes over the timescales examined for this study. We discuss why this assumption was made and the evidence in the literature that underpinned it in the following text.

The surface of the Moon is exposed to a surface bounded exosphere that is close to a vacuum. Microbes can survive exposure to the vacuum of space (*9*, *10*) (see additional references in the Introduction that document survival after exposure to open space) without experiencing more than a 10-fold decrease in the number of viable cells for timescales significantly longer than those relevant to this study. The combination of cold temperatures and exposure to vacuum (freeze-drying or lyophilization) is a common method used to preserve microorganisms for long-term storage on earth (*90*). This specifically includes studies that examined survival of exposure to vacuum for some of the microbes we examine, e.g., *Bacillus* (*8*, *12*) (in the latter study, a 1 log reduction took roughly 1000 days), *Deinococcus* (*20*), and *Aspergillus* (*21*, *22*) (although additional lower pressure studies have not yet been conducted). Given the timescales of exploration we are interested in or even longer periods such as the lunar synodic period, vacuum effects are unlikely to significantly inhibit survival.

Similarly, over the timescales of interest (1 day to 1 week), we assume that energetic particle radiation has a limited impact on the survivability of the microbes we are examining due to evidence in the literature regarding the weaker effect of such a mechanism. A broad examination of the effect of ionizing radiation on fungi found even 1 log reductions of included and relevant fungi and bacteria required nearly kilogray levels of ionizing radiation (*91*). In situ observations of energetic particle flux on the lunar surface (*92*) and in orbit near the Moon (*93*) indicate that, on the timescales of interest, total energetic particle dosage is likely to be many orders of magnitude below this. Recent research (*94*) into relevant energetic particle radiation dosages at the lunar south pole indicates radiation dosages roughly at the level of 10^−3^ gray (Gy) over a 30-day period during quiescent solar activity periods and ∼40 Gy for the most energetic coronal mass ejection (CME) events recorded (based on the highly energetic 1989 CME event). Recent research indicates a significantly less than 1 log reduction in wild-type strains of *Aspergillus* given exposure to radiation dosages similar to the CME event using different types of energetic particle radiation (*18*). For exposure to radiation dosages relevant to quiescent sun exposure, experiments suggest there is barely any reduction in total population. The effect is observed to be similar for *Bacillus subtilis* based on experiments conducted that examined survivability given exposure to varying radiation dosages (*95*, *96*). Those studies also found a significantly less than 1 log reduction at the relevant radiation dosages. Total energetic particle flux including solar energetic particles result in lethal dose timescales of 100 s to 10,000 s of years, depending on assumptions related to solar energetic particle flux (lower times assume constant powerful CME associated fluxes). GCR fluxes are even lower than these solar-sourced energetic particle flux radiation dosages—based on the most recent in situ measurements from LRO CRaTER (*97*) and RADOM results on Chandrayaan 1 (*98*) (convolving fluxes with particle cross sections gives destruction timescales of more than 1 million years). Similarly, “Earth Wind” particles that affect the Moon from inside the Earth’s magnetosphere are much smaller in magnitude than solar energetic particles [with energy fluxes several orders of magnitude smaller (*99*), including during substorms (*100*)] and, consequently, similarly have relatively minimal effect on the survivability of the relevant microbes on the timescales we examine.

Low-temperature survival of microbes for lunar surface temperatures relevant to this study is supported by a substantial body of literature (see the Introduction for references of survival of microbes exposed to open space). Survival of microbes during exposure to such temperatures during space based experiments on survivability are cited in Introduction and provide direct evidence of the ability of relevant microbes to survive temperatures similar to those on the lunar poles. Dehydration techniques that preserve survivability at similar temperatures are also commonplace for relevant microbes (*90*). Last, a number of experimental studies have examined this survivability and found minimal to no effect on (and, in some cases, increased) the survivability of microbes at relevant lower temperatures (studies relevant to *Bacillus* and *Aspergillus* cited as an example) (*19*, *22*, *101*, *102*).

UV lethal dose values in Table 1 are calculated based on empirical studies referenced in the same table. For a combination of simplicity and due to lack of empirical studies that extend down to 6 log reductions, we use single-stage decay models based on empirical data to determine the fluence required for a lethal dose [D99.9999, 6 log reduction in the number of viable organisms (*8*, *103*)], which the medical community defines as functionally sterile (*104*)—this is approximately equivalent to a sterilization assurance level (SAL) (*105*) of 10^−6^. We use the empirical fluence value at the lowest survival rate given to determine the D99.9999 value so that we minimize the extent of extrapolation and use multiple points along the curve to fit the exponential decay. In the cases of *Aspergillus* (wild type) and *Deinococcus*, we use the fluence at 3 log reductions to extrapolate a lethal dose. For *Fusarium*, *Bacillus*, and *Staphylococcus*, we use D90-derived values to extrapolate lethal dose fluence.

We only choose inactivation studies that are conducted in air or on surfaces because inactivation fluence can vary significantly in studies conducted in a liquid medium due to attenuation effects. We also choose studies where experiments are conducted in dry air, given the potential influence of humidity on survival (*103*). In general, our choices to calculate UV lethal doses lead to underestimates of the likelihood of survival for the microbe, e.g., we will not be considering two-stage decay and shoulders in the decay rate curves—both features decreasing the susceptibility to UV radiation. In addition, we do not consider photoreactivation and photorecovery (*106*), as well as other repair mechanisms, for simplicity of analysis. Considering some of the timescales for reduction and the potential effectiveness of photorepair at low UV flux levels may be a factor increasing survivability and should be considered in future work. We choose microbe states—spores or vegetative states, based on the hardiest state for a particular microbe that was likely to exist in a human inhabited space and could consequently be transferred to the surface.

### UV and temperature calculation methodology

UV fluence and maximum temperatures for the polar region maps are derived from preexisting illumination and temperature maps for the south pole and north pole. For these regional maps, maximum summer temperatures from a previous work (*35*) (with a ground resolution of 240 m/pix) are compared to maximum growth temperature thresholds to ascertain survivability. The polar region maps of the UV fluence for the lunar south pole and north pole are created using the following methodology.

In these polar region maps, fractional illumination over a lunar synodic period for the lunar poles is given in a previous study (*36*) at with a resolution of 60 m/pix based on modeling of illumination conditions over a full 18.6-year lunar precession cycle. We combine the analytically [see equation 6 from (*55*)] integrated fractional illumination received at a particular location with the latitude-dependent UV flux to produce an estimate of the average instantaneous flux at each surface location. The analytical formulation (*55*) of illumination is based on orbital and latitudinal properties at a particular point on the surface, and we calculate the fluence in 0.05° latitudinal increments (∼1.5 km). The inclination of the Moon’s rotational axis relative to the Sun is set to 1.5424°, with calculations taken for “summer” periods to remain conservative regarding survivability. Bounds in equation 6 are based on longitudinal terminator values given variations in the rotational axis relative to the Sun and the time of the year. Although this does apply to the Moon, daily illumination values from a previous work (*36*) include this factor and have more details due to incorporation of topography and averaging over a lunar nutation cycle. As a result, bounds are taken as −90° to 90° longitude to have the normalized flux when incorporating the higher-detail fractional illumination values. These calculations also assume flat surfaces within the discrete element of calculation, which is relaxed in our later higher-resolution modeling. Once we obtained an integrated value, we then scale the total value of the fluence by the total fractional Sun visibility, which assumes equal parts day and night based on the longitudinal bounds.

The total fluence is then divided by the total time in the synodic period to obtain the averaged instantaneous flux—this is an approximate estimate of flux that can be used for daytime estimates of total flux over a period. Because this includes periods where a region is not illuminated, it will be an underestimate for integrated flux during times around noon for a location and will obviously be an overestimate during nighttime, dawn, and dusk periods. Despite these divergences at different times, this approximation is useful because it acts as a guidepost to making estimates of total UV fluence for a location and because accurate fluxes at higher spatial resolution are strongly influenced by local topographic effects, which are only calculated for our high-resolution analysis of sites. Operational, predictive estimates should be made using high-resolution models.

Regional and higher-resolution maps in Figs. 1 and 2, respectively, calculate direct illumination UV flux from a previous study (*107*) for wavelengths shortwards of 320 nm based on comparisons to previous works and reduced inactivation at longer wavelengths (*8*, *103*). Neglecting reflected light in these maps as a first-order approximation is a fairly accurate estimate given low albedos for most common minerals and surfaces at the lunar surface (plagioclase being a notable exception) (*108*–*110*) and the fact that scattered light is, at most, ∼1% of the total in illuminated regions (*111*). Time-dependent higher-spatial-resolution observations of different regions of the Moon (including those near PSRs) show that direct illumination is anywhere from three to five orders of magnitude greater than scattered light [five orders in figure 2 of (*112*) and roughly three orders in figure 8 of (*113*)]. However, as mentioned in the interpretation of Fig. 3, incorporating scattered light is necessary when probing PSRs within higher-resolution maps meant to inform exploration.

High-resolution maps in Figs. 2 and 3 model instantaneous surface UV fluxes for wavelengths shortwards of 320 nm also based on whole heliosphere interval (WHI) data (*107*) and incorporate a finite angular size for the Sun. Earthshine and all-sky stellar sources of light are not included due to their relatively low flux compared to solar sources. Although Earthshine can be comparable to scattered solar illumination in the most favorable circumstances (*114*) and may be worth including in higher-detail maps in specific cases, all-sky stellar sources are roughly three orders of magnitude weaker than scattered solar illumination (*115*) and would consequently operate on much longer timescales than relevant to this study. Illumination modeling is carried out by ray tracing on a LOLA-based topography of 5 m/pixel with the ShadowSpy tool (*57*), which leverages state-of-art ray-tracing libraries (*116*, *117*) and planetary ephemerides from the NAIF/Spice toolkit (*118*, *119*).

### Methodology of survivability based on maps

The global datasets mentioned previously were gathered for combined analysis into a geographical information system (GIS), using the ESRI ArcMAP software (ArcGIS Desktop 10.8.2), for the regional north polar and south polar maps. The base maps used for Fig. 1 for the polar regions are mosaics from the Lunar Reconnaissance Orbiter Camera (LROC) Wide Angle Camera (WAC), allowing a pixelated view of the data of 100 m/pix. Regional maps of survivability for the north pole and south pole mask the joint UV illumination and temperature maps for each microbe using the survivability criteria described previously (Table 1) integrated over relevant timescales. These regional maps provide a qualitative estimate of survivability due to the assumptions in the calculation of the UV fluence. The general effect of these assumptions is to provide an overestimate of fluence in regions that receive less illumination and an underestimate in well-illuminated regions—these variations are typically less than a factor of 3 but peak at an underestimate of roughly a factor of 8 in the most well-illuminated regions and an overestimate by a factor of 12 in the most poorly illuminated non-PSR regions. In rough descending order of importance, differences are due to (i) the flat terrain assumption used when applying equation 6 in a previous work (*55*). Fractional illumination values are often driven by significant local topography, which results in marked deviations of the local elevation angle of the Sun from the latitude-based flat terrain assumption. Because of the general low elevation angle for flat terrain, these differences can result in multiple factors divergences in total flux. (ii) Applying a fractional illumination scalar to the regional maps involves scaling a half-day/half-night illumination profile by the fractional value based on averaging over a full nutation cycle. However, elevation angle and, consequently, flux profiles are dependent on local elevation angle and fractional illumination values and therefore may diverge from this scaling. (iii) Longitudinal bounds in the integration of equation 6 in a previous work (*55*) are taken to be −90° and 90°, but these will be slightly different given the orbital and obliquity values and time of season. Although divergences in fluence versus higher-resolution maps may be large for specific places, these regional maps provide a good general guide for areas that may offer enhanced or diminished survivability—high-resolution maps should be used for accurate analysis of specific sites.

On the basis of the same dataset as Figs. 2 and 3, table S1 presents the fraction of surface within each site where different microbes are likely to survive for 24 hours after deposition, based on the cumulative UV flux received at the surface. As expected, UV flux depends on seasonality (which determines the elevation of the Sun above the local horizon) so that the extent of survivable areas is larger in the “local” winter than in summer. Because the cumulative UV flux is based on seasonal average instantaneous UV fluxes, the reported values might still change on a daily basis. The PSR column indicates the fraction of each site, which never receives direct sunlight during each season: Although bacteria are likely to have higher survivability rates in these areas, more detailed analyses are needed to assess the impact of UV light scattered from neighboring (illuminated) areas. As an example, we performed such analysis at the PSR within De Gerlache polar crater by considering scattered UV light (we assume an albedo of 0.1, a typical value for visible light on the Moon) from a region with a radius of 40 km around the PSR. Although scattered UV flux is ∼1% of the direct flux, only *Aspergillus* shows a widespread survivability within the PSR, thus highlighting the need for more detailed modeling. Table S2 shows the portion of each site where *Aspergillus* is expected to survive over time at different target sites of interest. One can note how, even after 5 to 7 Earth days, nonnegligible portions of the surface are still survivable after deposition in these regions where UV fluxes are relatively limited.
