# Supplementary material for: Potential survivable niches for microbial life on the lunar south pole
Source: Sci Adv. 2026 Aug 19;12(34):eaec0811. doi: 10.1126/sciadv.aec0811 (PMC13508803; doi:10.1126/sciadv.aec0811)
Supplement: Supplementary file 1 [file sciadv.aec0811_sm.pdf]

Science Advances
AAAS

Supplementary Materials for

Potential survivable niches for microbial life on the lunar south pole

Prabal Saxena *et al.*

Corresponding author: Prabal Saxena, prabal.saxena@nasa.gov

Sci. Adv. **12**, eaec0811 (2026)
DOI: 10.1126/sciadv.aec0811

This PDF file includes:

Tables S1 and S2

Table S1: **The percentages of the surface where each microbe can still survive after 24 hours of average UV flux.** The table shows the percentage of each region's surface where different microbes are likely to survive based on lethal doses from Table 1 and total UV flux received. The first column indicates the fraction of a region that is a PSR - this region would receive no direct sunlight and thus in this illumination scenario, all 5 microbes would be able to survive. The following columns indicate the non-PSR regions where the respective microbe would survive according to the above criteria. All of the rows without a * only model direct flux. The * indicates that both direct and scattered fluxes have been modeled and accounted for, which is why the PSR region is set to 0 since it is a recipient of scattered flux.

**Site**	**PSR Fraction**	***Aspergillus***	***Fusarium***	***Deinococcus***	***Staphylococcus***	***B. Subtili***
Spring/Autumn (Survivable Surface, %)						
Nobile Rim 2	15.71	12.51	3.18	2.59	1.12	0.97
Connecting Ridge	6.55	16.75	3.15	2.55	1.17	1.03
De Gerlache Rim	5.55	13.84	2.42	1.89	0.68	0.57
De Gerlache PSR*	0.00	40.19	1.35	0.56	0.00	0.00
Summer (Survivable Surface, %)						
Nobile Rim 2	10.81	9.23	1.45	1.03	0.33	0.28
Connecting Ridge	3.55	7.11	1.06	0.86	0.40	0.35
De Gerlache Rim	2.01	2.75	0.47	0.39	0.18	0.16
De Gerlache PSR*	0.00	25.39	0.03	0.01	0.00	0.00
Winter (Survivable Surface, %)						
Nobile Rim 2	23.18	15.43	3.04	2.37	0.91	0.79
Connecting Ridge	19.93	30.26	5.98	4.29	1.48	1.29
De Gerlache Rim	42.14	20.44	4.79	3.60	1.31	1.14
De Gerlache PSR*	0.00	49.12	3.80	1.92	0.11	0.06

Table S2: **Aspergillus survivability over extended time periods.** The table shows the percentage of survivable surface for *Aspergillus* after X Earth Days during Spring/Autumn in different regions using a similar comparison as the preceding table. The * indicates that both direct and scattered fluxes have been modeled and accounted for.

**Site**	**0.5 Days**	**1 Day**	**3 Days**	**5 Days**	**7 Days**
Nobile Rim 2	22.57	12.51	6.06	4.44	3.68
Connecting Ridge	31.29	16.75	6.55	4.54	3.67
De Gerlache Rim	24.85	13.84	5.39	3.66	2.88
De Gerlache PSR*	47.68	40.19	22.81	12.98	4.57
